# SARS-CoV-2 sero-immunity and quality of life in children and adolescents in relation to infections and vaccinations: the IMMUNEBRIDGE KIDS cross-sectional study, 2022

**DOI:** 10.1007/s15010-023-02052-5

**Published:** 2023-06-06

**Authors:** Geraldine Engels, Anna-Lisa Oechsle, Anne Schlegtendal, Christoph Maier, Sarah Holzwarth, Andrea Streng, Berit Lange, Andre Karch, Astrid Petersmann, Hendrik Streeck, Sabine Blaschke-Steinbrecher, Christoph Härtel, Horst Schroten, Rüdiger von Kries, Reinhard Berner, Johannes Liese, Folke Brinkmann, Nicole Toepfner, the IMMUNEBRIDGE KIDS study group, Johannes Forster, Oliver Kurzai, Franziska Pietsch, Elena Hick, Katharina Hecker, Thomas Lücke, Anna Hoffmann, Michaela Schwarzbach, Jakob Höppner, Denisa Drinka, Jakob Armann, Judith Blankenburg, Uta Falke, Josephine Schneider, Veronika Jäger, Viktoria Rücker, Manuela Harries, Max Hassenstein, Maren Dreier, Isabell von Holt, Axel Budde, Marc-André Kurosinski, Antonia Bartz, Gunnar Brandhorst, Melanie Brinkmann, Kathrin Budde, Marek Deckena, Marc Fenzlaff, Olga Hovardovska, Katja Kehl, Mirjam Kohls, Stefan Krüger, Kristin Meyer-Schlinkmann, Patrick Ottensmeyer, Jens-Peter Reese, Daniel Rosenkranz, Nicole Rübsamen, Mario Schattschneider, Christin Schäfer, Simon Schlinkert, Kai Schulze-Wundling, Stefan Störk, Carsten Tiemann, Henry Völzke, Theresa Winter, Peter Heuschmann, Matthias Nauck

**Affiliations:** 1 Department of Pediatrics, University Hospital Würzburg, Josef-Schneider-Straße 2, 97080 Würzburg, Germany; 2 Division of Epidemiology, Institute of Social Pediatrics and Adolescent Medicine, LMU Munich, Haydnstraße 5, 80336 Munich, Germany; 3 Children’s Hospital, Ruhr University of Bochum, Alexandrinenstrasse 5, 44791 Bochum, Germany; 4 Department of Pediatrics, Department of Pediatrics, Faculty of Medicine and University Hospital Carl Gustav Carus, Technische Universität Dresden, Dresden, Germany, Fetscherstr. 74, 01307 Dresden, Germany; 5 Helmholtz Centre for Infection Research (HZI), Inhoffenstraße 7, 38124 Brunswick, Germany; 6 German Centre for Infection Research (DZIF), TI BBD, Brunswick, Germany; 7 Institute for Epidemiology and Social Medicine, University of Münster, Albert-Schweitzer-Campus 1, 48149 Münster, Germany; 8 Institute for Clinical Chemistry and Laboratory Medicine, University Oldenburg, Rahel-Straus-Straße 10, 26133 Oldenburg, Germany; 9 Institute for Clinical Chemistry and Laboratory Medicine, University of Greifswald, Ferdinand-Sauerbruchstrasse, 17475 Greifwald, Germany; 10 Institute of Virology, University Hospital, University of Bonn, Venusberg-Campus 1, Gebäude 63, 53127 Bonn, Germany; 11 German Center for Infection Research (DZIF), Partner Site Bonn-Cologne, Brunswick, Germany; 12 Emergency Department, University Medical Center Göttingen, Robert-Koch Sr. 40, 37075 Göttingen, Germany; 13 Pediatric Infectious Diseases, Department of Pediatrics, Medical Faculty Mannheim, Heidelberg University, Theodor-Kutzer-Ufer 1-3, 68167 Mannheim, Germany; 14 Department of Pediatrics, University of Lübeck, Campus Lübeck, Ratzeburger Allee 160, 23538 Lübeck, Germany

**Keywords:** SARS-CoV-2, Omicron, Seroprevalence, Children and adolescents, Quality of life

## Abstract

**Purpose:**

The study evaluates the effects on sero-immunity, health status and quality of life of children and adolescents after the upsurge of the Omicron variant in Germany.

**Methods:**

This multicenter cross-sectional study (IMMUNEBRIDGE Kids) was conducted within the German Network University Medicine (NUM) from July to October 2022. SARS-CoV-2- antibodies were measured and data on SARS-CoV-2 infections, vaccinations, health and socioeconomic factors as well as caregiver-reported evaluation on their children’s health and psychological status were assessed.

**Results:**

497 children aged 2–17 years were included. Three groups were analyzed: 183 pre-schoolchildren aged 2–4 years, 176 schoolchildren aged 5–11 years and 138 adolescents aged 12–18 years. Positive antibodies against the S- or N-antigen of SARS-CoV-2 were detected in 86.5% of all participants (70.0% [128/183] of pre-schoolchildren, 94.3% of schoolchildren [166/176] and 98.6% of adolescents [136/138]). Among all children, 40.4% (201/497) were vaccinated against COVID-19 (pre-schoolchildren 4.4% [8/183], schoolchildren 44.3% [78/176] and adolescents 83.3% [115/138]). SARS-CoV-2 seroprevalence was lowest in pre-school. Health status and quality of life reported by the parents were very positive at the time of the survey (Summer 2022).

**Conclusion:**

Age-related differences on SARS-CoV-2 sero-immunity could mainly be explained by differences in vaccination rates based on the official German vaccination recommendations as well as differences in SARS-CoV-2 infection rates in the different age groups. Health status and quality of life of almost all children were very good independent of SARS-CoV-2 infection and/or vaccination.

**Trial registration:**

German Registry for Clinical Trials Identifier Würzburg: DRKS00025546 (registration: 11.09.2021), Bochum: DRKS00022434 (registration:07.08.2020), Dresden: DRKS 00022455 (registration: 23.07.2020).

**Supplementary Information:**

The online version contains supplementary material available at 10.1007/s15010-023-02052-5.

## Introduction

Since the emergence of the severe acute respiratory syndrome coronavirus type 2 (SARS-CoV-2) Omicron variant, the pandemic situation in Germany has fundamentally changed for children and adolescents. At the beginning of the pandemic, children contracted COVID-19 significantly less frequently and were less severely ill than adults [1–3]. In contrast, children and adolescents suffered markedly from pandemic restrictions such as social distancing and school closures. Several longitudinal surveys, such as the COPSY study (impact of COVID-19 on psychological health), found substantial psychological distress in 2/3 of all children and adolescents aged 7–17 years from direct and indirect consequences of the pandemic [4].

Since winter 2021/2022, the Omicron variant also spread rapidly in the pediatric population because of its increased transmissibility [5, 6]. Thus, during the Omicron surge, high SARS-CoV-2 seroprevalence rates were detected in children and adolescents in Germany (2–6 years: 70%, 14–17 years: 92% [6–8]), Switzerland (0–5 years: 76.7%, 6–11 years: 90.5% [9]), Italy (2–11 years: 57% [10]) and Ireland (1–4 years: 28.8%, 5–12 years: 43.5% [11]). However, compared with the Delta variant [12, 13], Omicron-associated illness was less severe and frequently asymptomatic, particularly in children younger than 5 years [14, 15]. Data from the USA furthermore suggested that the overall risk for infection and hospitalization was lower when considering the Omicron variant compared with earlier variants [16].

Since August 2021, the Standing Committee on Vaccination (STIKO) at the Robert Koch Institute recommended primary immunization against COVID-19 with two doses of the vaccine [17] and one booster vaccination for children and adolescents 12 years of age and older [18]. For children 5–11 years of age without preexisting conditions, one vaccine dose was recommended since May 2022 [19].

To assess past SARS-CoV-2 infections in children and adolescents—particularly during the spread of the Omicron variant, as well as the vaccination status—a multicenter, age-related SARS-CoV-2 seroprevalence study was conducted in the pediatric cohorts of the ad hoc project IMMUNEBRIDGE within the Network University Medicine (NUM) at three sites in Germany. In addition, parents were interviewed regarding the health and quality of life of their children.

The aim of this study was to assess the SARS-CoV-2 immune status and quality of life in the pediatric population 2 years after the onset of the COVID-19 pandemic. In particular, we aimed to detect potential age-related differences being of high importance for the future planning of preventive measures such as vaccination recommendation and testing strategies.

## Methods

**Fig. 1:**
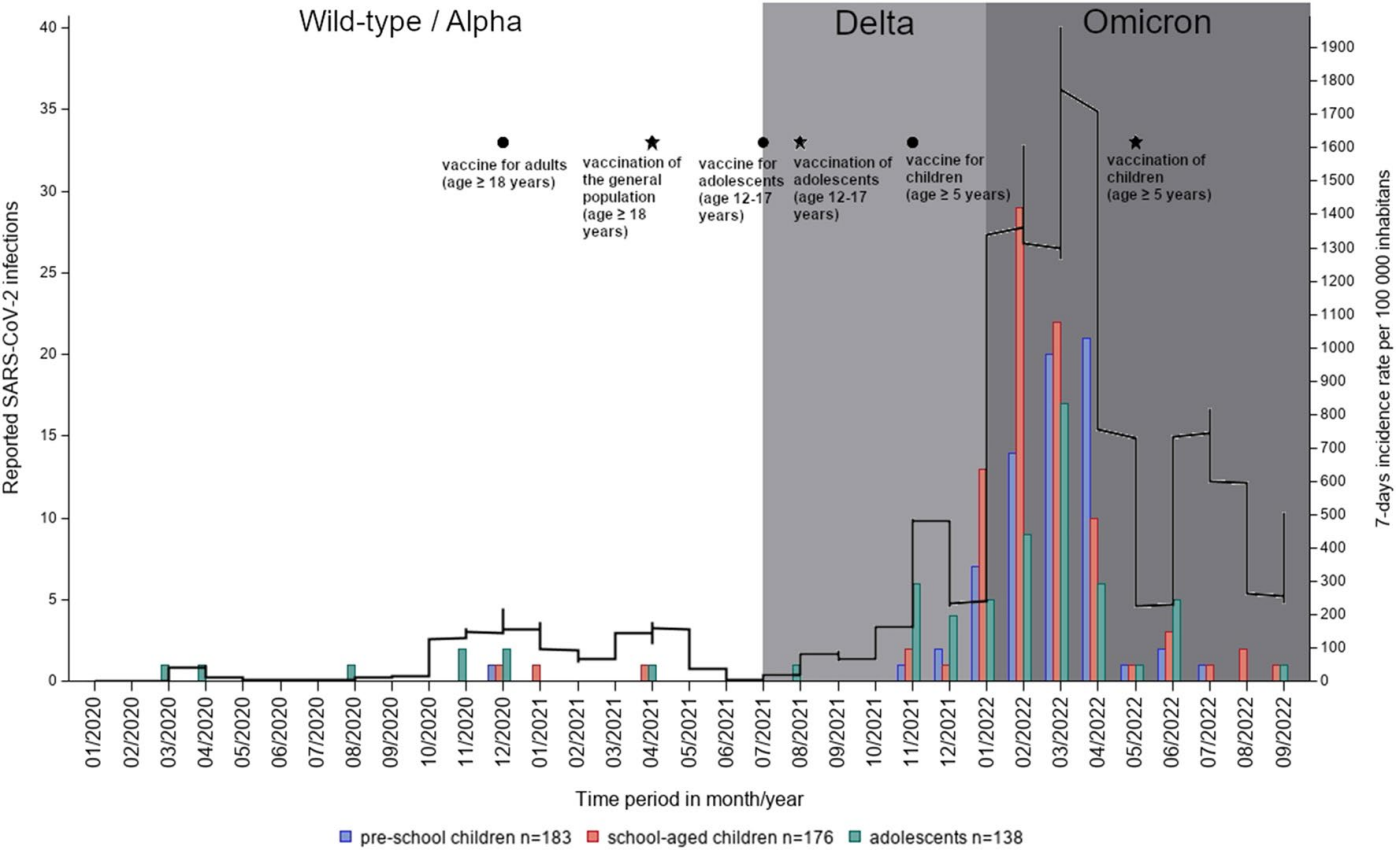
Dates and frequency of SARS-CoV-2 infections. Reported dates of SARS-CoV-2 infections by frequency and in the time course, stratified by age groups: pre-schoolchildren (blue), school-aged children (red) and adolescents (green). The SARS-CoV-2 virus variant that dominated in Germany during this time period is highlighted in different colors: wild-type- or Alpha-virus variant (white), Delta-virus variant (light gray) and Omicron-virus variant (dark gray). Dates and information on licensing of COVID-19 vaccines for the different age groups as well as 7 days SARS-CoV-2 incidence rates per 100,000 inhabitans in Germany are shown

From early July to mid-October 2022, antibodies against SARS-CoV-2 were determined at the participating sites in Würzburg, Bochum and Dresden. Blood was drawn by finger prick or venous blood sampling in children aged 2–17 years in a cross-sectional survey. Toddlers and pre-schoolchildren in Würzburg (2–6 years) were recruited from 9 out of 68 daycare centers (*n* = 275), which participated in two previous nonrandomized trails called “Wue-KiTa-CoV” [20] and “Wue-Kita-CoV-2” [21] with an enrollment rate of 34% (275/810 eligible children). Children in Bochum (2–11 years) were included as part of a follow-up study to the (Corona in Kids) CorKid seroconversion study (*n* = 145), which was carried out in 2148 children in 2020 in the Ruhr region (Western Germany) with an enrollment rate of 38% (145/377 eligible children) (Supplementary Information [SI] Fig. 1). In Dresden, adolescents (12–17 years) were recruited at two secondary schools (*n* = 107), one in the city of Dresden and one in the surrounding area, as amendment to the SchoolCoviDD19 study. Participation in the study was offered to the students and their caregivers of grade 8 and onwards. From these two schools, 107 eligible study participants were included in this analysis. The total cohort (*n* = 527) was stratified into three groups: 2–4 years (referred to as pre-schoolchildren), 5–10 years (referred to as schoolchildren) and 11–17 years (referred to as adolescents). Antibody determination was performed for all children and adolescents by immunological electrochemiluminescence assays (ECLIA, Roche Diagnostics GmbH, Mannheim, Germany). Quantitative measurement of SARS-CoV-2 spike (S)-protein antibodies (S-AB) occurring after both vaccination and disease (positive: values ≥ 0.8 binding antibody units (BAU)/ml) and qualitative measurement of nucleocapsid (N)-protein antibodies (N-AB) indicating past infection (positive: assay-specific cutoff index [COI]); values ≥ 1.0 COI) were performed. Demographic data, history of SARS-CoV-2 infection and vaccination status, general information on health, well-being and quality of life were assessed using a standardized health-related questionnaire recommended by the WHO [22–26]. The three cohorts reflect groups of healthy children in daycare centers and schools. Age-specific seroprevalence rates against the S- and N-antigen, number of self-reported infections, vaccinations and quality of life were reported with a 95% confidence interval. Adjustments for test quality were not made due to small expected differences at high seroprevalence rates. Potential differences between age groups were evaluated using the *X*^2^ test and Fisher’s exact test. Analyses were performed in SAS version 9.4 (SAS Institute, Cary, NC, USA). The study protocol was approved by the respective ethics committee at all three sites: Würzburg, reference 105/21; Bochum, reference BO-20/6927_7; Dresden, reference BO-EK-156042020. Written informed consent was obtained from all caregivers of the study participants.

## Results

### Study population

In total, the legal guardians of 497 participants (183 pre-schoolchildren (36.8%), 176 schoolchildren (35.4%) and 138 adolescents (27.7%)) consented to the study (54% female). An underlying condition was described in 11.0% (55/497) of the participants, the most common ones were allergies (4.8%, 24/497) and atopic dermatitis (3.4%, 17/497).

In 69% (344/497) of the children, Germany was given as the country of origin by both legal guardians. In 11% (57/497) of the participants, only one of the legal guardians reported Germany as the country of origin. In 7% (37/497), none of the legal guardians originated from Germany. Twelve percent (59/497) did not indicate their country of origin. Sixty-four percent noted grammar school as their highest school qualification. The median number of people living in a household was 4 (IQR 3.4).

### COVID-19 vaccination

Overall, 40.4% (201/497) of all study participants had received at least one COVID-19 vaccination. Of these, 4.4% (8/183) of pre-schoolchildren, 44.3% (78/176) of school-aged children and 83.3% (115/138) of adolescents had received at least one vaccination. Two vaccine doses were administered to 4 of the 8 vaccinated pre-schoolchildren (50.0%), 68 of the 78 vaccinated schoolchildren (87.1%) and 58 of the 115 vaccinated adolescents (50.4%). A booster vaccination had been received by 37.5% (3/8) of the vaccinated pre-schoolchildren, none of the 78 primary vaccinated schoolchildren and 43.4% (50/115) of the primary vaccinated adolescents (Table 1).

**Table 1:** COVID-19 vaccination status in the pediatric study population

Age groups	Vaccinated against COVID-19^a^	Number of vaccine doses in vaccinated study participants		
		1 COVID-19 vaccination	2 COVID-19 vaccinations	3 COVID-19 vaccination
Total	201/497 (40.4%)	18/201 (8.9%)	130/201 (64.6%)	53/201 (26.3%)
Pre-schoolchildren	8/183 (4.4%)	1/8 (12.5%)	4/8 (50.0%)	3/8 (37.5%)
School-aged children	78/176 (44.3%)	10/78 (12.8%)	68/78 (87.1%)	0/78 (−)
Adolescents	115/138 (83.3%)	7/115 (6.1%)	58/115 (50.4%)	50/115 (43.4%)

^a^Vaccinated at least once against COVID-19; absolute and relative frequencies overall or in the respective age group, shown as *n*/*N* (%), Pre-schoolchildren 2–4 years old, school-aged children 5–11 years old, adolescents 12–17 years old

### SARS-CoV-2 infections

History of a previous PCR-confirmed SARS-CoV-2 infection was reported in 47% (232/497) of all study participants: in 77 of 183 pre-schoolchildren (42.0%), in 90 of 176 schoolchildren (51.1%) and in 60 of 138 adolescents (43.5%).

Before January 2022, a SARS-CoV-2 infection was indicated in 4 of 77 pre-schoolchildren (5.2%), 2 of 90 schoolchildren (2.2%) and 19 of 65 adolescents (29.2%). Between January and April 2022, a PCR-confirmed SARS-CoV-2 infection was reported in 80.5% (62/77) of pre-schoolchildren, 82.2% (74/90) of schoolchildren and 56.9% (37/65) of adolescents (Fig. 1).

In the case of PCR-confirmed SARS-CoV-2 infections, 84.0% (195/232) of children and adolescents presented with symptoms of illness (pre-schoolchildren 76.6% [59/77], schoolchildren 84.4% [76/90] and adolescents 92.3% [60/65]). The number of children with unclear or absent symptoms was 23.3% (18/77) in pre-schoolchildren, 13.3% (12/90) in schoolchildren and 7.6% (5/65) in adolescents (Table 2). None of the study participants required hospitalization due to the SARS-CoV-2 infection.

**Table 2:** Self-reported PCR-confirmed SARS-CoV-2 infections

Study population	Reported PCR-confirmed infection	Reported symptoms	No/uncertain symptoms	No information on symptoms
Total	232/497 (46.7%)	195/232 (84.0%)	35/232 (15.0%)	2/232 (0.9%)
Pre-schoolchildren	77/183 (42.0%)	59/77 (76.6%)	18/77 (23.3%)	–
School-aged children	90/176 (51.1%)	76/90 (84.4%)	12/90 (13.3%)	2/90 (2.2%)
Adolescents	60/138 (43.5%)	60/65 (92.3%)	5/65 (7.6%)	–

Absolute and relative frequencies of all study participants with a history of symptoms in 232 PCR-confirmed SARS-CoV-2 infections in total or, respectively, in the age groups shown as *n*/*N* (%)

### SARS-CoV-2 seroprevalence

Among the 138 adolescents, 136 had detectable SARS-CoV-2 S-AB or N-AB. The proportion of adolescents with S-AB or N-AB, which are usually detected after infection and/or vaccination, was 98.5% (136/138). In adolescents, the proportion of N-AB detected only after SARS-CoV-2 infection was 67.4% (93/138). Ninety-four percent (166/176) of school-aged children had detectable S-AB (93.1%, 164/176) or N-AB (73.8%, 130/176), and 69.9% (128/183) of pre-schoolchildren had detectable S-AB (68.3%, 125/183) or N-AB (60.6%, 111/183) (Table 3).

**Table 3:** Seroprevalence of SARS-CoV-2

Study population	Seroprevalence of antibodies against the spike protein and/or against the nucleocapsid protein	Antibodies against the spike protein	Antibodies against the nucleocapsid protein
Total	430/497 (86.5%)	425/497 (85.5%)	334/497 (67.2%)
Pre-schoolchildren	128/183 (70.0%)	125/183 (68.3%)	111/183 (60.7%)
School-aged children	166/176 (94.3%)	164/176 (93.2%)	130/176 (73.9%)
Adolescents	136/138 (98.6%)	136/138 (98.6%)	93/138 (67.4%)
(a) Study participants vaccinated against COVID-19^a^			
Total	201/201 (100%)	201/201 (100%)	122/201 (60.7%)
Pre-schoolchildren	8/8 (100%)	8/8 (100%)	4/8 (50%)
School-aged children	78/78 (100%)	78/78 (100%)	46/78 (59.0%)
Adolescents	115/115 (100%)	115/115 (100%)	72/115 (62.6%)
(b) Study participants not vaccinated against COVID-19			
Total	225/292 (77.1%)	220/292 (75.3%)	208/292 (71.3%)
Pre-schoolchildren	120/175 (68.6%)	117/175 (66.9%)	107/175 (61.1%)
School-aged children	86/96 (89.6%)	84/96 (87.5%)	82/96 (85.4%)
Adolescents	19/21 (90.5%)	19/21 (90.5%)	19/21 (90.5%)

Prevalence of antibodies against the SARS-CoV-2 spike protein and nucleocapsid protein in total and stratified by age groups and vaccination status

^a^Vaccinated at least once against COVID-19. Absolute and relative frequencies in total and, respectively, in the age groups, shown as *n*/*N* (%)

Of the vaccinated study participants, SARS-CoV-2 antibodies were detectable in all participants (S-AB 100% [201/201], N-AB 60.7% [122/201]). SARS-CoV-2 seroprevalence among nonvaccinated pre-schoolchildren, schoolchildren and adolescents was 68.9% (120/175), 89.6% (86/96) and 90.5% (19/21), respectively.

### Seroprevalence in relation to vaccination against and/or infection by SARS-CoV-2

**Fig. 2:**
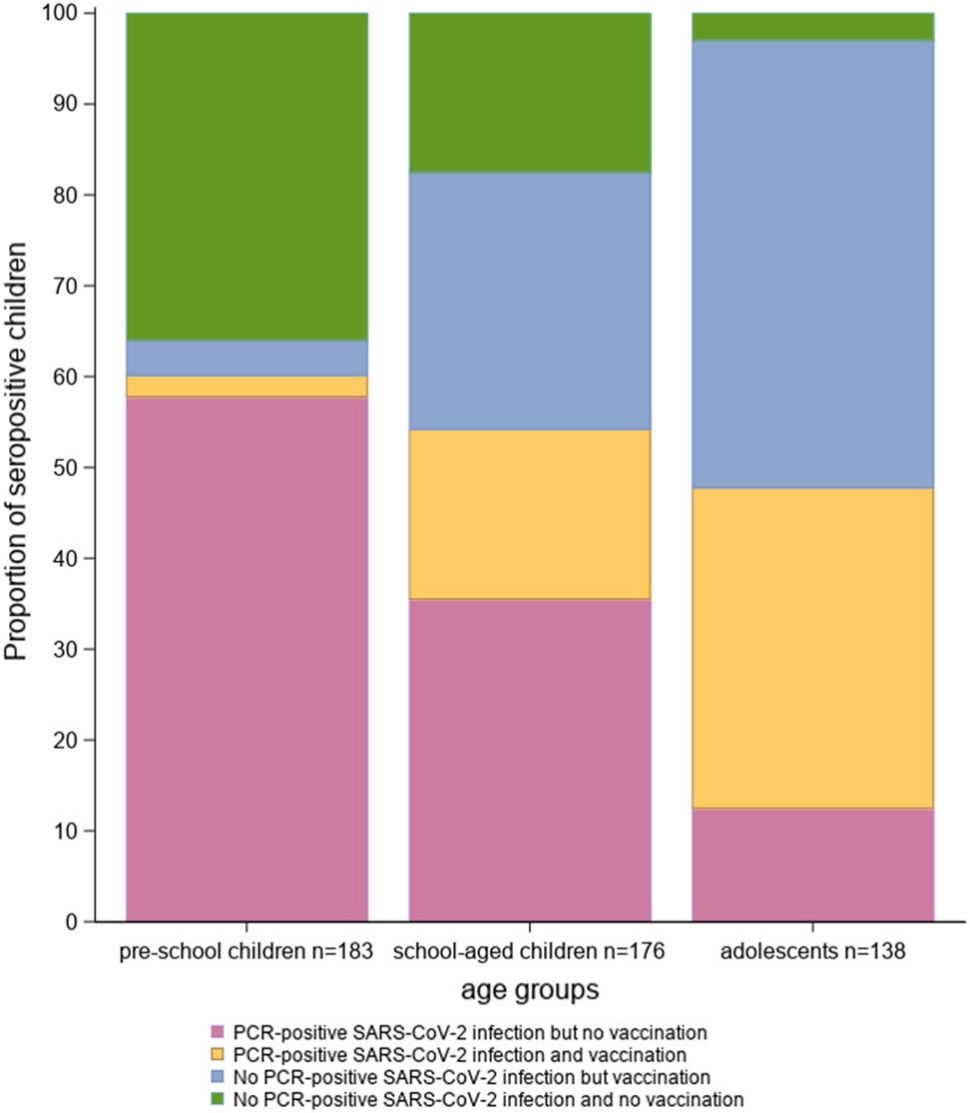
Age-dependent differences in SARS-CoV-2 seroprevalence associated with SARS-CoV-2 infection and/or COVID-19 vaccination. SARS-CoV-2 seropositive study participants by COVID-19 vaccinations and PCR-confirmed SARS-CoV-2 infections (yellow), seropositive by vaccinations without PCR-confirmed SARS-CoV-2 infections (blue), seropositive by PCR-confirmed SARS-CoV-2 infections without vaccination (red) and seropositive without vaccinations and without PCR-confirmed SARS-CoV-2 infections (green); stratified by age groups (128 pre-schoolchildren, 166 schoolchildren, 136 adolescents). *PCR* polymerase chain reaction

Among seropositive study participants, 2.3% (3/128) of pre-schoolchildren, 18.6% (31/166) of schoolchildren and 35.2% (48/136) of adolescents were vaccinated against COVID-19 and had a history of PCR-confirmed SARS-CoV-2 infection. COVID-19 vaccination without PCR-confirmed infection was described by 3.9% (5/128) of pre-schoolchildren, 28.3% (47/166) of schoolchildren and 49.2% (67/136) of adolescents. No COVID-19 vaccination, but a PCR-confirmed SARS-CoV-2 infection was reported by 57.8% (74/128) of pre-schoolchildren, 35.5% (59/166) of schoolchildren and 12.5% (17/136) of adolescents. Thirty-six percent (46/128) of pre-schoolchildren, 17.4% (29/166) of schoolchildren and 2.9% (4/136) of adolescents reported no COVID-19 vaccination and no PCR-confirmed SARS-CoV-2 infection (Fig. 2, SI Table 1).

### *N*-antibody seroprevalence in children without PCR-confirmed SARS-CoV-2 infection

Fifty-three percent (265/497) of all study participants had no PCR-confirmed SARS-CoV-2 infection since the beginning of the COVID-19 pandemic: 57.9% (106/183) in the pre-schoolchildren group, 48.8% (86/176) in schoolchildren and 52.8% (73/138) in adolescents. The proportion of children and adolescents in this group with N-AB, but no PCR-confirmed SARS-CoV-2 infection was 40.5% (43/106) of pre-schoolchildren, 52.3% (45/86) of schoolchildren and 39.7% (29/73) of adolescents.

### Health status and quality of life

At the time of survey in June–October 2022, parents described their children’s health as well as quality of life as “excellent” (38.8%, 193/497 and 38.4%, 191/497) or “very good” (38.4%, 191/497 and 32.1%, 160/497) in all age groups. There were no differences between children and adolescents without COVID-19 vaccination or PCR-confirmed SARS-CoV-2 infection (134/497, including 41.0% (55/134) reporting “excellent” and 38.8% (52/134) reporting “very good”) compared to children and adolescents with COVID-19 vaccination and/or PCR-confirmed SARS-CoV-2 infection (364/497, including 38.1% (139/364) reporting “excellent” and 48.3% (176/364) reporting “very good”, *p* = 0. 17). Also, there were no differences between age groups and between vaccinated and unvaccinated schoolchildren and adolescents. There were also no differences in the group of pre-schoolchildren (*p* = 0.14) between the 55 SARS-CoV-2 seronegative (38.2%, 21/55 indicating “excellent” and 36.6%, 20/55 indicating “very good”) and 46 SARS-CoV-2 seropositive study participants (36.9%, 17/46 indicating “excellent” and 52.2%, 24/46 indicating “very good”).

## Discussion

In summer/early fall 2022, children and adolescents in Germany demonstrated a high SARS-CoV-2 seroprevalence of 70–99% [6, 9, 15, 27]. Surveys of adults, however, indicated even higher SARS-CoV-2 seroprevalence rates [28–31]. However, within the pediatric population, the SARS-CoV-2 seroprevalence rates showed marked differences between the different age groups: among schoolchildren and adolescents, SARS-CoV-2 antibodies are detectable in almost every child, whereas among pre-schoolchildren, SARS-CoV-2 antibodies are detectable in only 70%. In adolescents, this can partially be explained by the fact that COVID-19 vaccination has been generally recommended in Germany since August 2021. At least one vaccination dose was reported by 85% of adolescents in our study population compared to 69.4% of all adolescents in statewide vaccination records in Germany [32]. In schoolchildren, our cohort presented a significantly lower vaccination rate (45%), but still clearly above the vaccination rate of 19.9% at the national level [32]. The lower vaccination rate of schoolchildren in comparison to adolescents is probably due to the fact that the general vaccination recommendation by the STIKO for this age group was not available until May 2022 [19] and many schoolchildren at that time had already experienced a laboratory-confirmed SARS-CoV-2 infection.

For children under 5 years of age, the vaccines Comirnaty^®^ and Spikevax^®^ were approved in October 2022 by the European Medicines Agency (EMA) [33]. However, the STIKO in Germany recommends a vaccination only for children with certain underlying diseases since November 2022 [34].

There were no relevant differences between the three age groups in the frequency of PCR-confirmed SARS-CoV-2 infections. Of note, approximately one-third of non-vaccinated young children had SARS-CoV-2 antibodies without a history of a symptomatic or PCR-confirmed SARS-CoV-2 infection. However, oligo- or asymptomatic SARS-CoV-2 infections in children with detectable anti-N antibodies decreased markedly with increasing age: thus, only 3% of unvaccinated adolescents compared with 17% of schoolchildren and 36% of pre-schoolchildren had no history of PCR-confirmed and/or symptomatic SARS-CoV-2 infection, although N-AB were detectable. Most SARS-CoV-2 infections were mild and none of the study participants required hospitalization due to a SARS-CoV-2 infection. The three cohorts in this study included mostly healthy children attending daycares and schools, with the objective to assess seroprevalence against SARS-CoV-2. Therefore, the study cannot assess the rare risk of hospitalization and complications of COVID-19 in children.

Contrary to the pronounced psychological stress for children and adolescents during the pandemic shown in other surveys [4, 35, 36], parents of study participants reported generally a very good or excellent health and quality of life in summer/autumn 2022, 2 years after the onset of the COVID-19 pandemic. No relevant differences were reported depending on whether a SARS-CoV-2 infection or a COVID-19 vaccination had occurred. The positive statements on health status and quality of life may be explained by the normalized daily routine for children and adolescents at the time of the survey, with kindergartens and schools open, and the decrease of most restrictions in daily life. However, it has to be taken into account that a large proportion of the study participants came from households with a high socioeconomic status, which is regarded as a protective factor against psychological stress [4, 37, 38].

Limitations of our study include regional restriction to three locations in Germany, no children younger than 2 years of age included and, in addition to the aforementioned high socioeconomic status, the potential selection of more cautious and concerned parents who might have been more willing to participate in such a study. The latter can also be assumed due to the comparatively high vaccination rate, which was clearly above the national average, especially in the schoolchildren group. Furthermore, the study represents healthy children and does not specifically consider vulnerable groups.

## Conclusion

The high SARS-CoV-2 sero-immunity rates among children and adolescents in Germany indicate that by fall 2022, large proportions of children and adolescents were exposed to the virus or viral antigens through infection and/or vaccination.

Age-dependent SARS-CoV-2 seroprevalence differences are evident, which are partly due to different infection or vaccination rates in the various age groups. Here, the largely unvaccinated cohort of pre-schoolchildren showed a 70% SARS-CoV-2 seropositivity with a relatively high proportion of unnotified and undiagnosed SARS-CoV-2 infections. With a very positive health status and quality of life reported in all age groups in summer/autumn 2022, no significant negative effects on the psychological well-being appear to result from the age-dependent differences in immunity acquisition following SARS-CoV-2 infections or vaccinations in children and adolescents.

### Supplementary Information

Below is the link to the electronic supplementary material.

Supplementary file1 (DOCX 17 KB)

## Data Availability

Data are partially available via https://zenodo.org/record/6968574 and https://zenodo.org/record/7177592. Further data are available on reasonable request from the authors.
