# Supplementary material for: SARS-CoV-2 sero-immunity and quality of life in children and adolescents in relation to infections and vaccinations: the IMMUNEBRIDGE KIDS cross-sectional study, 2022
Source: Infection. 2023 Jun 6;51(5):1531–9. doi: 10.1007/s15010-023-02052-5 (PMC10243264; doi:10.1007/s15010-023-02052-5)
Supplement: Supplementary file 1 — Supplementary file1 (DOCX 17 KB) [file 15010_2023_2052_MOESM1_ESM.docx]

**SUPPLEMENTORY MATERIAL**

**INFECTION - A Journal of Infectious Diseases**

**SARS-CoV-2 sero-immunity and quality of live in children and adolescents in relation to infections and vaccinations: The** **IMMUNEBRIDGE KIDS cross-sectional study, 2022**

Geraldine Engels MD, PhD ^1^, Anna-Lisa Oechsle , MSc ^2^, Anne Schlegtendal MD^3^, Christoph Maier MD^3^, Sarah Holzwarth MD^4^, Andrea Streng PhD^1^, Berit Lange MD, MSc^5,6^, Andre Karch MD, MSc^7^, Astrid Petersmann MD^8,9^, Hendrik Streeck MD^10,11^, Sabine Blaschke-Steinbrecher MD^12^, Christoph Härtel MD^1^, Horst Schroten MD^13^, Rüdiger von Kries MD^2^, Reinhard Berner MD^4^, Johannes Liese MD, MSc^1^, ^#^Folke Brinkmann MD ^3,14^, ^#^Nicole Toepfner MD^4^ on behalf of the IMMUNEBRIDGE KIDS study group

^#^Co-senior authors

**Correspondence to:**

Dr. Nicole Töpfner, MD

Department of Pediatrics, University Hospital and Medical Faculty Carl Gustav Carus, Technische Universität Dresden

Fetscherstr. 74, 01307 Dresden, Germany, Tel: 0351 458 2440

E-Mail: Nicole.Toepfner@uniklinikum-dresden.de

| **Status of reported** **SARS-CoV-2 infection and/or COVID-19 vaccination.** | **Number of Study participants** | **Percent** | **Lower 95% Confindence Limit** | **Upper 95% Confindence Limit** |
| --- | --- | --- | --- | --- |
| **Pre-school children n=183** | | | | |
| PCR-positive SARS-CoV-2 infection but no vaccination | 74 | 57.8 | 49.2 | 66.4 |
| PCR-positive SARS-CoV-2 infection and vaccination | 3 | 2.3 | 0.0 | 5.0 |
| No PCR-positive SARS-CoV-2 infection but vaccination | 5 | 3.9 | 0.5 | 7.3 |
| No PCR-positive SARS-CoV-2 infection and no vaccination | 46 | 35.9 | 27.6 | 44.3 |
| **School-aged children n=176** | | | | |
| PCR-positive SARS-CoV-2 infection but no vaccination | 59 | 35.5 | 28.2 | 42.9 |
| PCR-positive SARS-CoV-2 infection and vaccination | 31 | 18.7 | 12.7 | 24.6 |
| No PCR-positive SARS-CoV-2 infection but vaccination | 47 | 28.3 | 21.4 | 35.2 |
| No PCR-positive SARS-CoV-2 infection and no vaccination | 29 | 17.5 | 11.7 | 23.3 |
| **Adolescents n=138** | | | | |
| PCR-positive SARS-CoV-2 infection but no vaccination | 17 | 12.5 | 7.0 | 18.1 |
| PCR-positive SARS-CoV-2 infection and vaccination | 48 | 35.3 | 27.2 | 43.4 |
| No PCR-positive SARS-CoV-2 infection but vaccination | 67 | 49.3 | 40.8 | 57.7 |
| No PCR-positive SARS-CoV-2 infection and no vaccination | 4 | 2.9 | 0.1 | 5.8 |
